# Sub-MHz homogeneous linewidth in epitaxial Y_2_O_3_: Eu^3+^ thin film on silicon

**DOI:** 10.1515/nanoph-2024-0682

**Published:** 2025-02-14

**Authors:** Diana Serrano, Nao Harada, Romain Bachelet, Anna Blin, Alban Ferrier, Alexey Tiranov, Tian Zhong, Philippe Goldner, Alexandre Tallaire

**Affiliations:** 129667 Chimie ParisTech, PSL University, CNRS, Institut de Recherche de Chimie Paris, F-75005 Paris, France; INL, Université de Lyon, Ecole Centrale de Lyon, CNRS UMR 5270, 69134 Ecully, France; Faculté des Sciences et Ingénierie, Sorbonne Université, UFR 933, Paris, France; Pritzker School of Molecular Engineering, University of Chicago, 60637, Chicago, IL, USA

**Keywords:** thin film, rare-earth, homogeneous linewidth, quantum technologies

## Abstract

Thin films provide nanoscale confinement together with compatibility with photonic and microwave architectures, making them ideal candidates for chip-scale quantum devices. In this work, we propose a thin film fabrication approach yielding the epitaxial growth of Eu^3+^ doped Y_2_O_3_ on silicon. We combine two of the most prominent thin film deposition techniques: chemical vapor deposition (CVD) and molecular beam epitaxy (MBE). We report sub-megahertz optical homogeneous linewidths up to 8 K for the Eu^3+^ dopants in the film, and lowest value of 270 kHz. This result constitutes a ten-fold improvement with respect to previous reports on the same material, opening promising perspectives for the development of scalable and compact quantum devices containing rare-earth ions.

## 1 Introduction

Rare-earth ions (REI) are among the most extensively studied optical centers in solid-state systems [1]. Yet, leveraging the exceptional optical and spin coherence properties of REI for quantum information technologies is a relatively recent and rapidly evolving field [2], [3]. Thanks to advances in photonic technologies, on-chip rare-earth quantum devices such as single-photon sources [4], quantum memories [5], quantum transducers [6] and simulators [7], are progressively emerging, offering performance capabilities that could be complementary to, or even surpass, other solid-state quantum technologies. Despite these advancements, most nanophotonic quantum devices still rely on bulk REI-doped materials [4], [5], [6], which may present challenges for future scalability and functionality. To address this limitation, one promising approach currently being explored is the direct integration of REIs into nanophotonic structures [8], [9]. Another interesting approach to achieving scalability together with compactness is by embedding REIs in a thin-film directly grown onto a silicon platform [10], [11]. This configuration allows for integration with photonic components alongside electronics that can address the REI spin states. For optimal performance, the film should ideally be single crystalline to minimize defects and therefore reduce static and dynamic contributions to their optical and spin linewidths. Epitaxial films are also of interest to achieve a uniform response from all ions to polarized light, and to external electric and/or magnetic fields. Besides, to maximize the quantum capabilities of the embedded REI, the crystalline material constituting the film should present low or zero nuclear and electron spin density in order to limit optical and spin dephasing due to magnetic noise.

Binary oxides such as Y_2_O_3_ have been established as promising crystal host candidates to fulfill the previously mentioned criteria [12], [13], [14], [15]. The dominating nuclear spin bath contribution in Y_2_O_3_ comes from yttrium, estimated at just a few hundred of Hz for the optical transition of Eu^3+^ at 580 nm. This low magnetic noise was evidenced by the near-radiatively-limited optical homogeneous linewidth of 760 Hz exhibited by a bulk Y_2_O_3_: Eu^3+^ single crystal [16]. Growth of epitaxial Y_2_O_3_ thin films on silicon has been reported using deposition techniques such as molecular beam epitaxy (MBE) [11] and pulsed layer deposition (PLD) [17], [18]. MBE-grown Y_2_O_3_ films, for instance, can achieve excellent material quality [11]. An optical homogeneous linewidth of 3 kHz has been recently reported for Er^3+^ ions in the C_3*i*_ site of an MBE-grown Y_2_O_3_ thin film [19]. A versatile and cost-efficient alternative to the previously mentioned thin film deposition methods is chemical vapor deposition (CVD). CVD allows for obtaining Y_2_O_3_ films with thicknesses varying from few nm to several μm, and for a full range of REI doping concentrations [20]. Yet, so far reported CVD Y_2_O_3_ films are polycrystalline [20], [21], [22], yielding homogeneous linewidth values of several MHz for Eu^3+^ ions occupying the C_2_ [22].

In the present work we propose a thin film fabrication approach yielding the epitaxial growth of Y_2_O_3_ on silicon. This approach combines two of the most prominent thin film deposition techniques: chemical vapor deposition (CVD) [20] and molecular beam epitaxy (MBE) [23]. We investigate the optical coherence properties of Eu^3+^ ions doped in the film and observe narrow optical homogeneous linewidths down to 270 kHz. Those were assessed by spectral hole burning investigations carried out at different excitation powers and temperatures. This result constitutes a ten-fold improvement with respect to previous reports on polycrystalline Y_2_O_3_: Eu^3+^ CVD thin films [20], [22], and the lowest homogeneous linewidth reported so far for Eu^3+^ in a nanoscale film. It also ranks among the narrowest optical homogeneous linewidths ever observed for REI in oxide thin films [19], [24].

## 2 Results

### 2.1 Epitaxial growth

**Figure 1:**
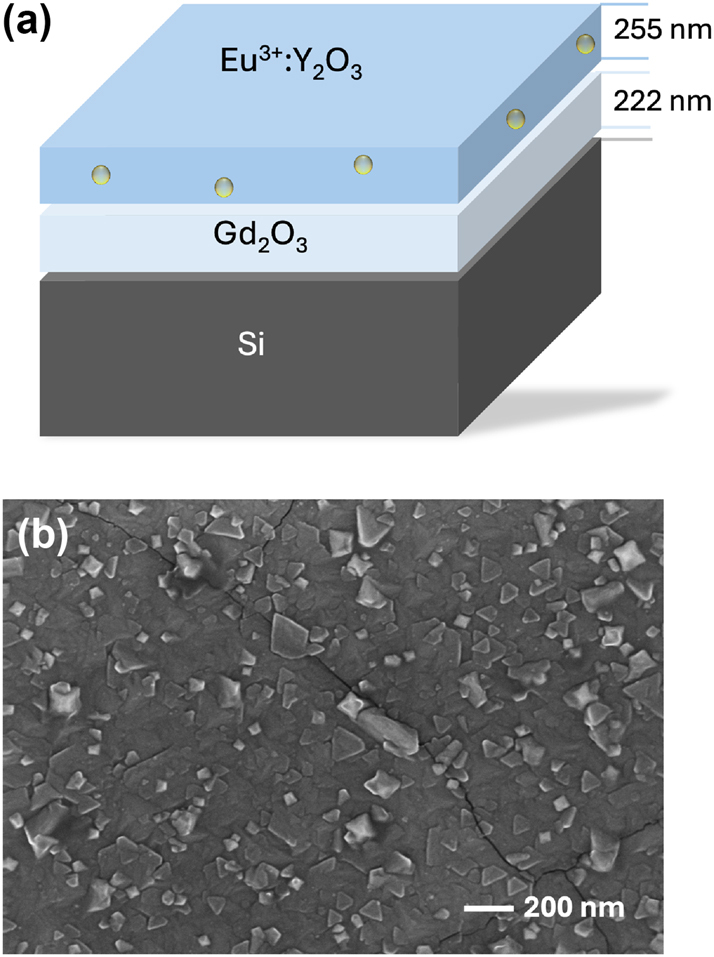
Epitaxial Y_2_O_3_: Eu^3+^ thin film on Gd_2_O_3_/Si substrate. (a) Schematic view of the multilayer structure. (b) Y_2_O_3_: Eu^3+^ film surface morphology observed by scanning electron microscopy (SEM).

Y_2_O_3_: Eu^3+^ (2 % Eu^3+^) was deposited by direct liquid injection chemical vapor deposition (DLI-CVD) on a commercial Si(111) wafer presenting a MBE-grown Gd_2_O_3_ film on top. Gd_2_O_3_ constitutes an excellent template due to a low lattice mismatch with Si (around 0.44 %), allowing for being epitaxially grown by MBE on this substrate [25]. The Gd_2_O_3_ film used in this investigation has a thickness of about 225 nm as confirmed by ellipsometry analysis (see Figure S1). Besides, XRD analysis confirmed that it is epitaxial on silicon with a mosaicity of about 0.24° around the [111] direction (see Figure S2). The multilayer structure is schematically represented in Figure 1(a). The home-built DLI-CVD reactor is described in detail in Ref. [20]. Beta-diketonate Y and Eu complexes (YTHD and EuTHD) with 3N purity were used as precursors. They were dissolved in mesitylene (99 % purity), flown into two independent injectors and vaporized at 200 °C. The vapors were carried to the reaction chamber by N_2_, and O_2_ was also flown to deposit the film. Deposition occurred at a pressure of 30 mbar with the substrate heated at 800 °C.

In the described deposition conditions, the epitaxial growth of Y_2_O_3_ directly on silicon is impaired by the unavoidable oxidation of the silicon wafer surface. This leads to the formation of an amorphous SiO_2_ superficial layer on top of which Y_2_O_3_ tends to grow in a polycrystalline manner [20], [22]. To overcome this limitation, we took advantage here of the Gd_2_O_3_ buffer layer, grown by MBE under conditions that avoid the formation of an amorphous oxide interface. The MBE-grown Gd_2_O_3_ buffer layer provides in addition a low-lattice-mismatch (1.9 %) template for the subsequent epitaxial growth of Y_2_O_3_: Eu^3+^ in our CVD reactor.

**Figure 2:**
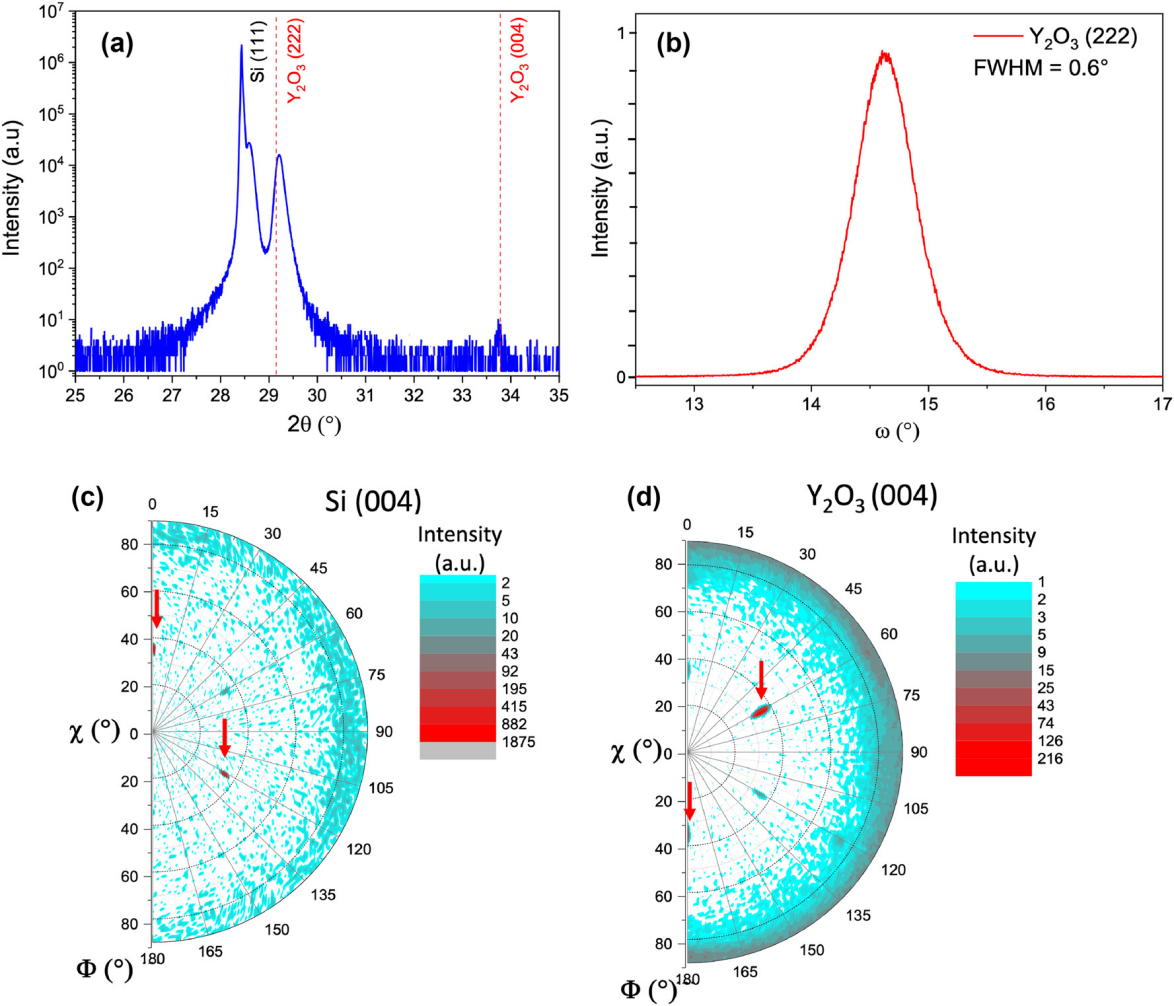
High-resolution X-ray diffraction (XRD) investigations. (a) *θ*/2*θ* scan showing [111] out-of-plane orientation for the Y_2_O_3_:Eu^3+^ film. (b) Omega scan around one of the (222) diffraction peaks yielding a mosaicity of 0.6°. (c, d) Pole figures showing in-plane orientation corresponding to that of the substrate, further confirming epitaxial growth. The arrows indicate the position of the diffraction spots in the reciprocal space.

The as-deposited Y_2_O_3_ film on Gd_2_O_3_/Si was observed under a scanning electron microscope (SEM) showing a rather uniform and smooth surface (Figures 1(b) and S4). A few square-shaped grains stand out, presenting sizes between 50 and 200 nm and a few cracks possibly related to thermal mismatch with the Si substrate. This surface morphology is in clear contrast with that of previously reported polycrystalline Y_2_O_3_ films [20], [22], of much rougher appearance, and with clearly distinguishable faceted grains and grain boundaries. The epitaxy of the Y_2_O_3_: Eu^3+^ film on the Gd_2_O_3_/Si template was confirmed by high-resolution X-ray diffraction analysis. *θ*/2*θ* scans were taken at different angles (Figures 2(a) and S3) evidencing single [111] out-of-plane orientation for the Y_2_O_3_: Eu^3+^ film, similar to that of the intermediate Gd_2_O_3_ layer and also of the Si(111) substrate (Figure 2(a)). A mosaicity of the order of 0.6° is also observed (Figure 2(b)), a reasonable value for an oxide film on silicon [26]. Definitive confirmation of the film epitaxy is given by the pole figures (Figure 2(c) and (d)), showing a single in-plane orientation of the Y_2_O_3_: Eu^3+^ thin film, therefore confirming single-domain epitaxy on Si.

### 2.2 Eu^3+^ emitters properties

**Figure 3:**
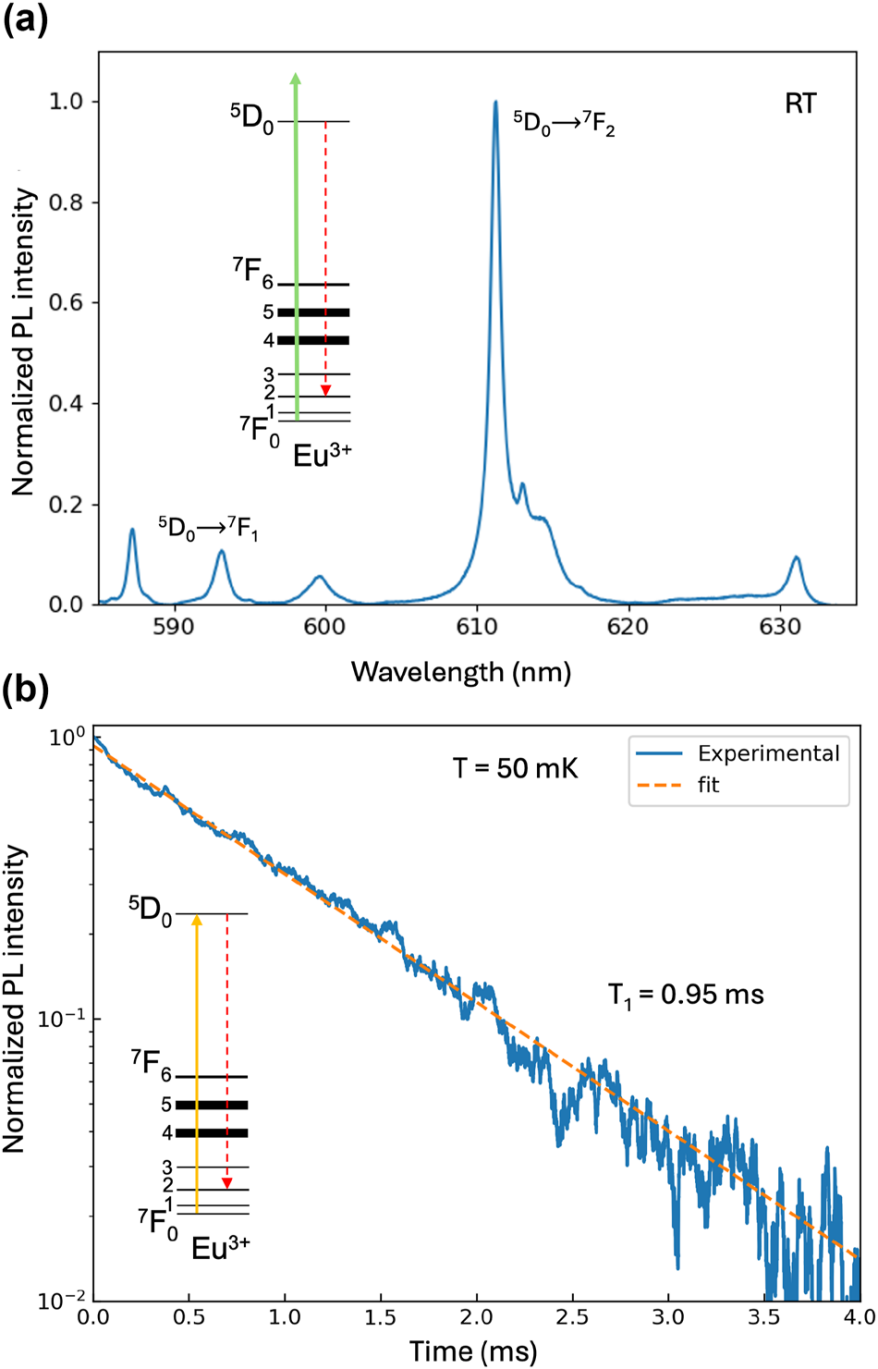
Photoluminescence (PL) spectroscopy of Eu^3+^ ions in the Y_2_O_3_ thin film. (a) Room temperature PL spectrum showing several Eu^3+^ emission lines including the strong ^5^D_0_ →^7^F_2_ emission at 611 nm. (b) ^5^D_0_ →^7^F_2_ PL decay recorded at 50 mK and 611 nm under resonant ^7^F_0_ →^5^D_0_ excitation at 580.79 nm. The dashed orange line corresponds to a single exponential fit to the data yielding a population lifetime *T*_1_ = 0.95 ms for the ^5^D_0_ excited state.

The optical properties of Eu^3+^ ions embedded in the epitaxial film were investigated in view of their use as quantum emitters. Prior to the optical characterizations, the film was annealed in air for 2 h at 950 °C to cure defects in the aim of reducing the emitters homogeneous linewidth [22]. The efficient incorporation of Eu^3+^ dopants into the well-crystallized Y_2_O_3_ film was confirmed by analyzing the photoluminescence (PL) emitted by the sample under 532 nm excitation at room temperature. A strong emission peak at 611 nm was observed, corresponding to the Eu^3+^ ^5^D_0_ →^7^F_2_ emission line in cubic Y_2_O_3_ (Figure 3(a)) [22]. This emission spectrum presents no difference with respect to that measured for the as-deposited film (see Figure S5), confirming the absence of parasitic phases that could originate from surface or interfacial diffusion during the annealing [22].

For probing the emission dynamics and optical homogeneous linewidth of Eu^3+^ ions, the thin film was mounted into a dilution refrigerator equipped with a home-built optical microscopy setup and excited using a tunable dye laser with a 300 kHz linewidth. Full experimental details are given in the Supplementary Materials (SM) document (Section 4). The lifetime (*T*_1_) of the ^5^D_0_ excited state was then measured at 50 mK by resonantly exciting the ^7^F_0_ →^5^D_0_ transition at 580.79 nm (Figure 3(b)). A value of 0.95 ms was obtained by single-exponential adjustment, comparable to bulk Y_2_O_3_: Eu^3+^ radiative lifetime [27]. This result highlights the good crystalline quality and low defect content of the film, and establishes a lower limit to the optical homogeneous linewidth of 170 Hz (1/2*πT*_1_).

**Figure 4:**
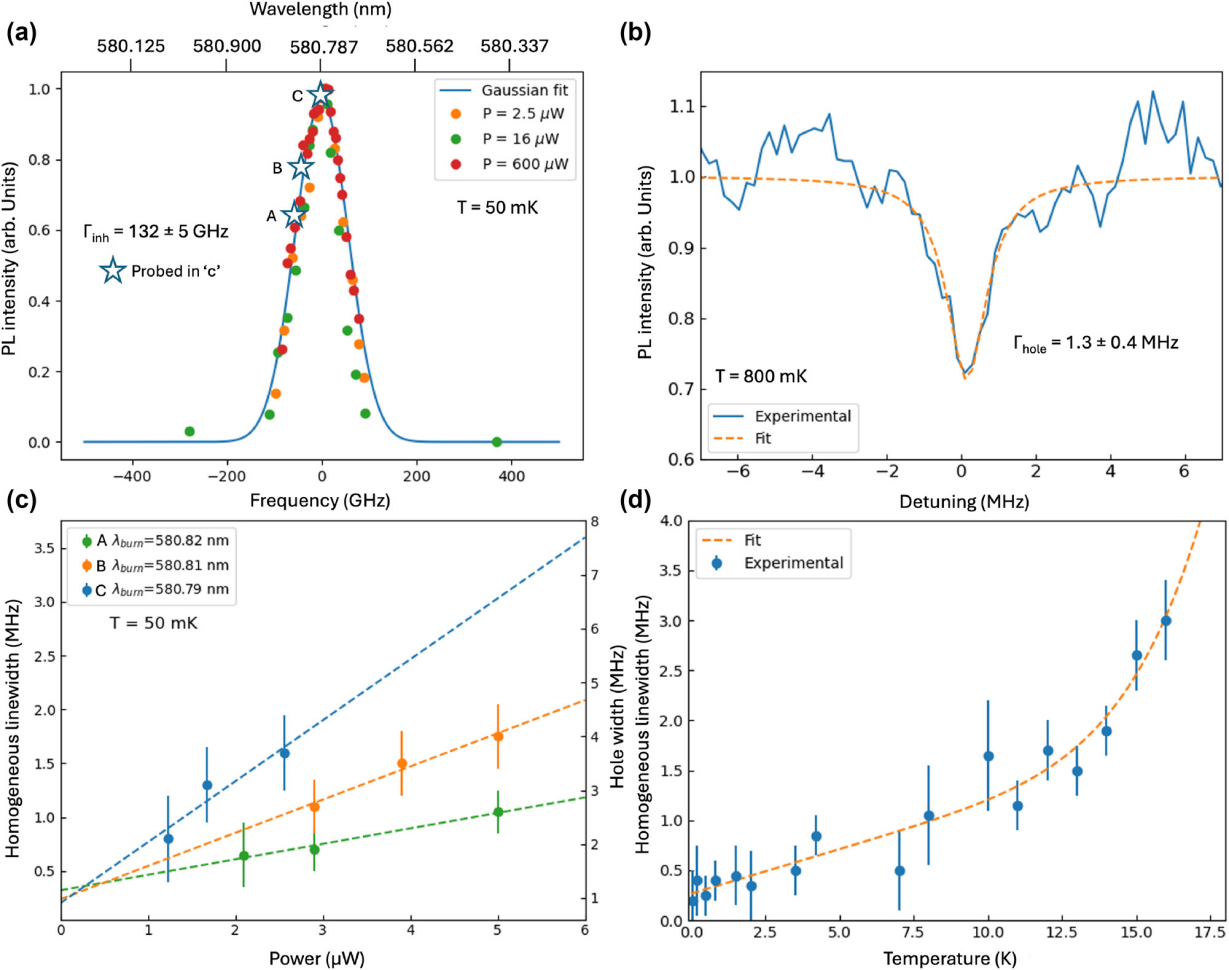
High resolution and coherent optical characterizations at mK temperature. (a) Inhomogeneous linewidth for the ^5^D_0_ →^7^F_0_ transition measured at three different excitation powers by scanning the excitation wavelength while monitoring the ^5^D_0_ →^7^F_2_ emission at 611 nm. A Gaussian curve fit to the data yields a full width at half maximum (FWHM) of 132 ± 5 GHz. The stars indicate spectral position within the linewidth of the ions probed in (c). (b) Spectral hole burned at 580.787 nm at a temperature of 800 mK (blue solid line). The dashed orange line correspond to a Lorentzian curve fit to the data yielding a hole width of 1.3 ± 0.4 MHz. (c) Homogeneous linewidth (left axis) and hole width (right axis) as a function of burn power measured at three different frequencies within the inhomogeneous line (see (a)). (d) Homogeneous linewidth as a function temperature (blue points). The dashed orange line corresponds to a fit to these data using eq. (1).

The optical inhomogeneous line of the ^7^F_0_ ↔^5^D_0_ transition is displayed in Figure 4(a). We observe a full width at half maximum (FWHM) of 132 GHz by monitoring the PL emission intensity at 611 nm as a function of the excitation wavelength. The measurement was carried out at three different excitation powers to rule out power broadening effects. The measured inhomogeneous linewidth is significantly broader than that expected for a nominal concentration of 2 %, which would be less than 40 GHz. This expectation is based on previous spectroscopic studies conducted on high-quality bulk Y_2_O_3_: Eu^3+^ samples with varying doping concentrations [28], assuming a linear trend with increasing concentration [29]. The measured linewidth of 132 GHz is comparable to that observed in annealed polycrystalline Y_2_O_3_: Eu^3+^ thin films with the same Eu^3+^ concentration (2 % at.) deposited via DLI-CVD. This broadening reveals the presence of a substantial number of static defects in the film, likely attributed to strain induce by the annealing treatment [22]. The 2 % Eu^3+^ concentration spectrally distributed over the 132 GHz lead to an average of ∼60 emitters per excitation bandwidth (300 kHz) in a diffraction-limited excitation volume. The intensity of the detected PL signals indicates however that we are collecting photons from at least 10^3^ ions which we attribute to a larger excitation volume and power broadening effects (see Section 5 discussion in SM).

The optical homogeneous linewidth (Γ_*h*_) exhibited by the Eu^3+^ ions in the film was finally probed by persistent spectral hole burning. Spectral holes presenting a narrow width of the order of 1 MHz were recorded (Figure 4(b)), a value which is one order of magnitude lower than spectral holes measured at 3 K in polycrystalline CVD Y_2_O_3_: 2 % Eu^3+^ thin films [20], [22]. This result suggests that the epitaxial ordering achieved in the present CVD film goes along with a significantly lower decoherence probably due to a lesser amount of defects [30]. To gain a further insight into the dynamical mechanisms limiting Γ_*h*_, spectral holes were measured at different spectral positions within the inhomogeneous line (labelled A, B and C and indicated by star symbols in Figure 4(a)) and also using different excitation powers. The obtained hole widths (Γ_hole_) and resulting homogeneous linewidths (Γ_*h*_), estimated as Γ_hole_/2-Γ_laser_, with Γ_laser_ = 300 kHz, are displayed in Figure 4(c). A linear Γ_*h*_ broadening is observed when increasing power, but with a slope that decreases when the excitation wavelength is moved away from the center of the inhomogeneous line (C at 580.79 nm) towards the side (A at 580.82 nm). In other words, the observed power broadening effect is stronger when the excited ion ensemble increases, revealing an excitation-induced contribution to Γ_*h*_ in the Y_2_O_3_: Eu^3+^ film proportional to the number of excited ions [31]. Yet, a power-independent Γ_*h*_ between 200 and 300 kHz can be extrapolated for all probed excitation wavelengths at the zero power limit, showing no significative differences in the optical coherence properties of different ion ensembles within the inhomogeneous line. The evolution of Γ_*h*_ with temperature was also measured and is given in Figure 4(d). The obtained Γ_*h*_(*T*) was fitted by

(1) $$\Gamma_{h}\left(T\right)=\Gamma_{0}+\alpha T+\beta T^{7}$$

where Γ_0_ represents the temperature-independent contribution to Γ_*h*_, *α* is the coupling constant to the so-called two-level systems (TLS), or dynamical disorder modes characterized by a linear temperature dependence [32], and *β* the coupling constant to two-phonon Raman scattering processes [29]. Best-fit parameters to the data yield Γ_0_ = 270 ± 87 kHz, *α* = 89 ± 14 kHz/K and *β* = 5 ± 1 × 10^6^ kHz/K^7^. The TLS contribution, indicative of the amount of dynamic disorder, is here 5 times lower than in previously reported polycrystalline CVD Y_2_O_3_: 2 % Eu^3+^ thin films [22] while comparable to that observed in optimized Y_2_O_3_: Eu^3+^ (0.3 % at.) nanoparticles of 100 nm size also probed by spectral hole burning (*α* = 132 kHz/K) [32].

## 3 Discussion and conclusions

In this manuscript, we report a method to obtain rare-earth doped Y_2_O_3_ thin films with enhanced optical properties for quantum technology applications. Due to a low lattice mismatch to Y_2_O_3_ (1.9 %), and the possibility to grow it by MBE with high crystalline quality, epitaxial Gd_2_O_3_ on Si offers an ideal buffer layer that could be overgrown by our active Y_2_O_3_:REI layer using CVD. This hybrid MBE-CVD approach allows preventing unwanted oxidation of the Si substrate while still benefiting from the simplicity and verstatility of CVD to grow complex thin film architectures. This approach could be extended to other epitaxial buffer oxide layers on Si, including Y_2_O_3_ itself, which can be also grown by MBE [11], [33], while offering the perfect lattice matching for the DLI-CVD growth [11], [33]. The only strong requirement is a good quality MBE buffer to succeed in the epitaxy. This means that the buffer itself must epitaxial and present a smooth surface. Our approach can also be extended to other REIs. In particular, Er^3+^ offers an optical transition in the telecom band directly compatible with Si photonic circuits (PCs) [4]. The transition wavelength of Eu^3+^, on the other hand, can be addressed using other photonic architectures such as Si_3_N_4_ resonators on top of the CVD film [34]. Coupling to tunable Fabry–Perot fiber cavities would be also possible [35], [36], by removing all or part of the Si substrate [19].

In conclusion, by the proposed hybrid CVD-MBE thin film fabrication approach, we are indeed combining the best features and advantages of both techniques. This is in particular evidenced by the significant step forward in terms of optical coherence properties achieved in this work for the active Y_2_O_3_: Eu^3+^ thin film, showing an optical homogeneous linewidth as narrow as 270 kHz assessed by persistent spectral hole burning. This accounts for a ten-fold improvement with respect to previous reports on the same material, opening promising perspectives for further developments in the field of quantum-grade REI thin films.

## Supplementary Material

Supplementary Material Details
