# Supplementary material for: Sub-MHz homogeneous linewidth in epitaxial Y2O3: Eu3+ thin film on silicon
Source: Nanophotonics. 2025 Feb 14;14(11):1809–15. doi: 10.1515/nanoph-2024-0682 (PMC12133248; doi:10.1515/nanoph-2024-0682)
Supplement: Supplementary file 1 — Supplementary Material Details [file j_nanoph-2024-0682_suppl_001.pdf]

Supplementary Materials

D. Serrano, N. Harada, R. Bachelet, A. Blin, A. Ferrier, A. Tiranov, T. Zhong, P. Goldner, and A. Tallaire

Sub-MHz homogeneous linewidth in epitaxial Y_2O_3: Eu^{3+} thin film on silicon

1 Spectroscopic ellipsometry

Spectroscopic ellipsometry (Woollam iSE) in the range 400-1000 nm was used to evaluate the thicknesses of the Gd_2O_3 buffer and Y_2O_3 active layers. The optical refractive index of Gd_2O_3 and Y_2O_3 were obtained using a Cauchy model. (Figure S1). A thin SiO_2 interlayer was considered in the fit. The thickness of this interlayer is estimated to 3 nm, and it did not have any impact on the epitaxial growth of Gd_2O_3 on the Si substrate. This contrasts with interfacial SiO_2 layers formed during direct CVD on Si, of several tens of nm that prevent direct epitaxy.

2 X-ray diffraction

A high-brilliance X-ray diffractometer (SmartLab from Rigaku, equipped with a 9 kW rotating anode and a two-bounce Ge(220) monochromator) was used to investigate the crystalline orientation of both the substrate and deposited CVD film. $\theta/2\theta$ and ω scans of the substrate prior to deposition are displayed in Figure S2. It shows that the Gd_2O_3 buffer layer on Si is epitaxial with a low mosaicity. $\theta/2\theta$ scans of the sample after CVD deposition are displayed in Figure S3. The absence of diffraction peaks corresponding to other directions than [111] for the Y_2O_3: Eu^{3+} thin film confirm the epitaxial growth. A residual remaining of the 004 peak can be observed in the logarithmic scale plot in Figure S2-left, suggesting the presence of remaining defects.

3 Scanning electron microscopy

The scanning electron microscopy (SEM) image of the surface of the films was taken with with a field emission gun (FEG-SEM) ZEISS Merlin microscope. The Gd_2O_3/Si substrate was imaged before the CVD deposition and is displayed in Figure S4-left). The MBE-grown Gd_2O_3 film presented a very smooth surface in which triangular grains can be distinguished, characteristic of a (111) morphology. In addition, the image shows that the crystalline domains seem to have the same in-plane orientation, as expected from an epitaxial film. Figure S4-right displays the Y_2O_3: Eu^{3+} thin film grown on top of the Gd_2O_3 by CVD. A rather smooth surface is also observed for the later, as well as triangular grains. A few squared grains can also be observed, due to some residual but very limited (100) growth (Figure S3).

4 Optical spectroscopy

The Eu^{3+} emission spectrum at room temperature was recorded using a Renishaw InVia apparatus with a 50× objective and a 532 nm laser as the excitation source. Eu^{3+} emission spectra taken from the film after deposition and after annealing at 950 °C for 2 h are displayed in Figure S5.

Low-temperature high-resolution and coherent measurements were carried out on a Bluefors SD dilution refrigerator. An aspheric lens (Edmund Optics, NA = 0.77) mounted on a nano-positioner (Attocube) was used to focus the excitation into the film and and to collect the Eu^{3+} emission. A low-pass fluorescence filter was used to filter-out the Eu^{3+} emission at 611 nm corresponding to the $^5D_0 \rightarrow {}^7F_2$ (Figure 3(a) in main text). A high-sensitivity photomultiplier tube (PMT) was used as detector (Hamamatsu R10699). The excitation around 580.78 nm ($^7F_0 \leftrightarrow {}^5D_0$ transition) was provided by a continuous wave (CW) dye laser (Matisse DS) with linewidth of ∼300 kHz. This linewidth is taken as the effective excitation bandwidth in subsequent calculations. Pulsed sequences were created by an acousto-optic modulator (AOM) in double pass configuration (Figure S2-top) and driven

by an arbitrary waveform generator (AWG). All optical experiments were carried out without applying an external magnetic field.

The spectral hole burning sequence consisted of a burning pulse of 1 ms duration and ~1-10 μW power followed by a readout pulse with a duration of 200 μs. The waiting time between burn and readout pulses was 10 ms, i. e. significantly longer than the optical T_1 to make sure that the probed hole is indeed a persistent spectral hole and not a transient one (Figure S6). The readout pulse was followed by 5 frequency-sweeping pulses (over 200 MHz with the same power used for the burning pulse, omitted in Figure S2-bottom for simplicity) to reset the ground-state nuclear-spin population back to thermal equilibrium. The full sequences was repeated with a rate of 10 Hz, and the PL decay signal following the readout pulse was averaged over 1000 shots to improve signal to noise ratio. The decreased population of the ground-state levels pumped by the burning pulse leads to a decreased emission intensity following the readout pulse. The full hole profile can therefore be reconstructed by monitoring the PL intensity following the readout pulse. In practice, the frequency of the burning pulse was kept fixed at ν_0 while the readout frequency was varied by an amount $\pm\Delta\nu$ around ν_0 (Figure S6). It is important to note that the alternative often used to detect persistent spectral holes in REI crystals, i.e. monitoring the laser transmission as a function of readout frequency is here very challenging to implement due to the very low optical depth of the 200 nm film. The PL-detection approach is indeed much more sensitive for thin samples. Background fluorescence emissions, which could artificially decreased the hole contrast were filtered out using a time-resolved detection, taking advantage of the long optical T_1 of Eu^{3+} ions, over several ms, with respect to background species, decaying in the first 100s of μs following the readout pulse. The $^5D_0$ photoluminescence (PL) decay and $^5D_0 \leftrightarrow {}^7F_0$ inhomogeneous linewidth were recorded with the same experimental setup used for the spectral hole burning measurements.

5 Number of emitters

The efficiency of the detection in Figure S6, including the PMT, the oscilloscope used to record the signals, and a coupling resistance of 100 kΩ, was calibrated using an attenuated laser beam at 1.24×10^{-11} V/W. Typical PL signals detected in our SHB experiments following the readout pulse are of the order of few mV. This would correspond to a few thousand emitters (e.g. 760 for 1 mV and 2280 for 3 mV), considering a total collection efficiency estimated at 7%. The later accounts for the collection efficiency of the high NA (0.77) lens in front of the sample, and for losses in the different optical elements, including the beam splitter and the low-pass filter. The branching ratio of the detected transition $^5D_0 \rightarrow {}^7F_2$ (~70%) is also considered. This number of emitters is significantly higher than that expected per single excitation bandwidth in a diffraction limited volume, with a focal diameter estimated using the Airy approximation for a Gaussian beam ($d = 0.9\lambda/NA \sim 700$ nm), which is around 60 emitters. This estimation also takes into account the experimental inhomogeneous line (132 GHz), and the Eu^{3+} density in site C_2 (2.8×10^{20} ions/cm^3). We attribute the mismatch between these two values to two main factors : a sub-estimation of the effective excitation bandwidth due to power broadening (Figure 4(c)) together with a sub-estimation of the focus diameter by the diffraction limit approximation. A focus of the order of 3 μm would indeed yield a number of emitters comparable to that derived from the detected signal. We have indeed a non-negligible experimental incertitude concerning the focus diameter as the focal distance was empirically optimized to maximize the collected PL emission intensity. Other factors contributing to the mismatch could be an over-estimated collection efficiency and/or detection sensitivity. Despite the incertitude derived from the different assumptions and some possible empirical errors, these estimations clearly show that our PL-detection-based methodology is suitable for probing small ensembles of ions in nanoscale materials.

References

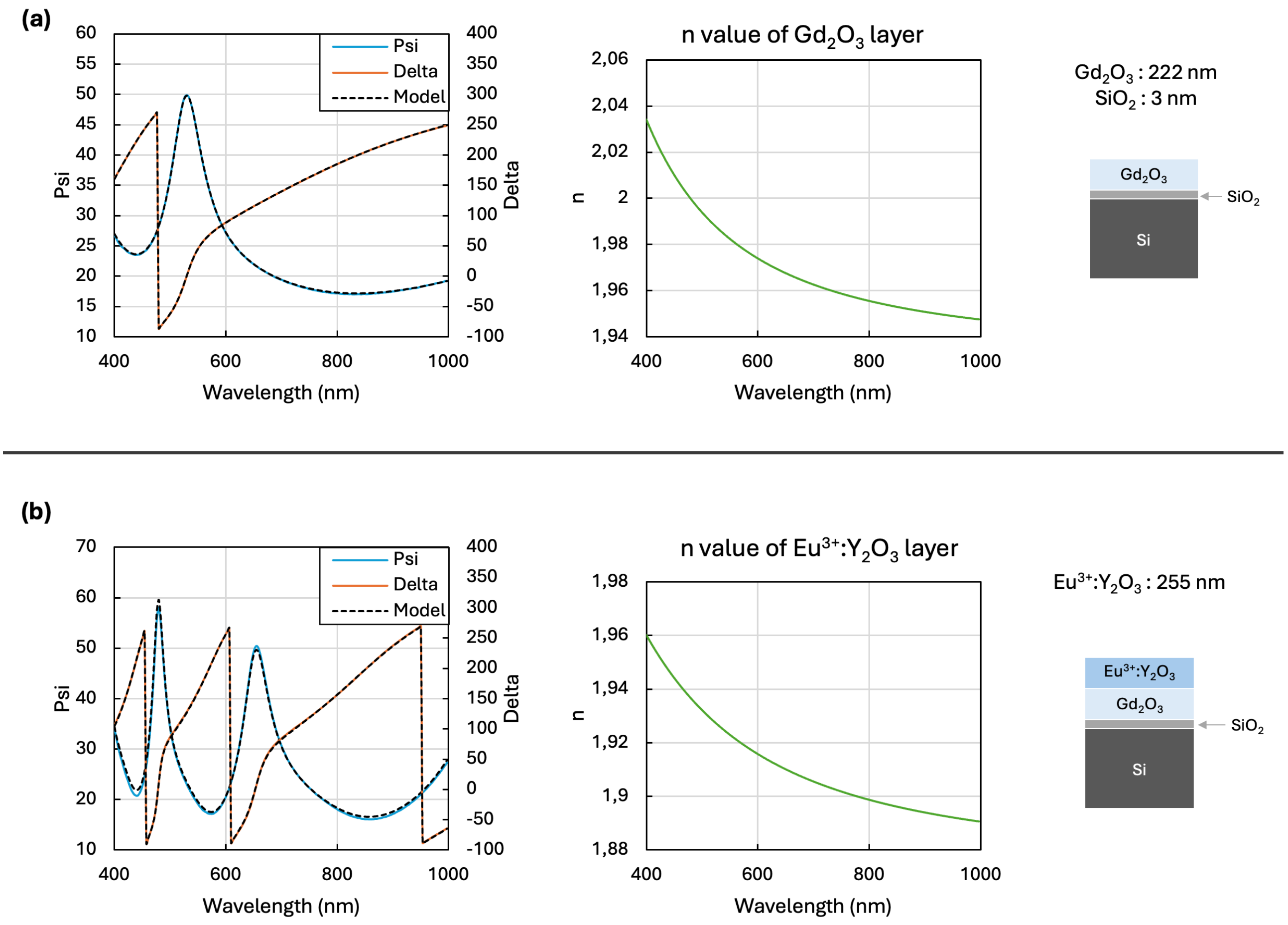

Fig. S 1: Spectroscopic ellipsometry analysis of the (a) Gd_2O_3 buffer layer on Si and (b) Y_2O_3: Eu^{3+} thin film grown on the buffer layer by CVD, yielding thicknesses of 222 nm and 255 nm respectively. Psi and Delta stand for the ellipsometric angle and phase difference, while the result of the Cauchy model is displayed as dashed lines on the graphs. The refractive indexes of the 2 oxide layers extracted from the model are also presented. A thin SiO_2 interlayer was considered in the fit.

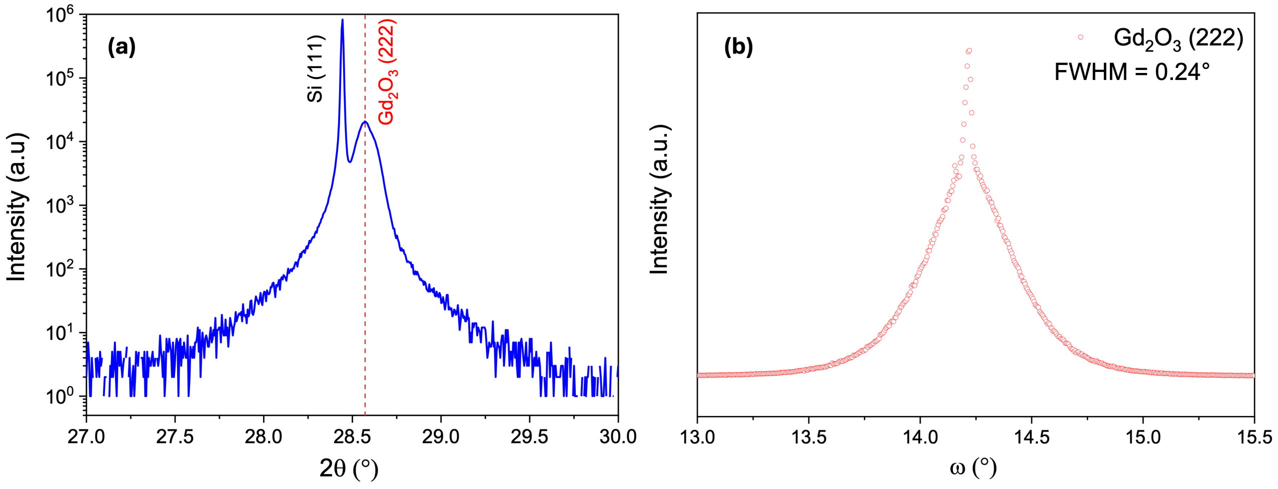

Fig. S 2: XRD characterization of the MBE-grown Gd_2O_3 buffer layer on Si used for CVD deposition. (a) $\theta/2\theta$ scan showing a single [111] out-of-plane orientation of the oxide film. (b) ω scan around the (222) diffraction peak showing a FWHM of 0.24°.

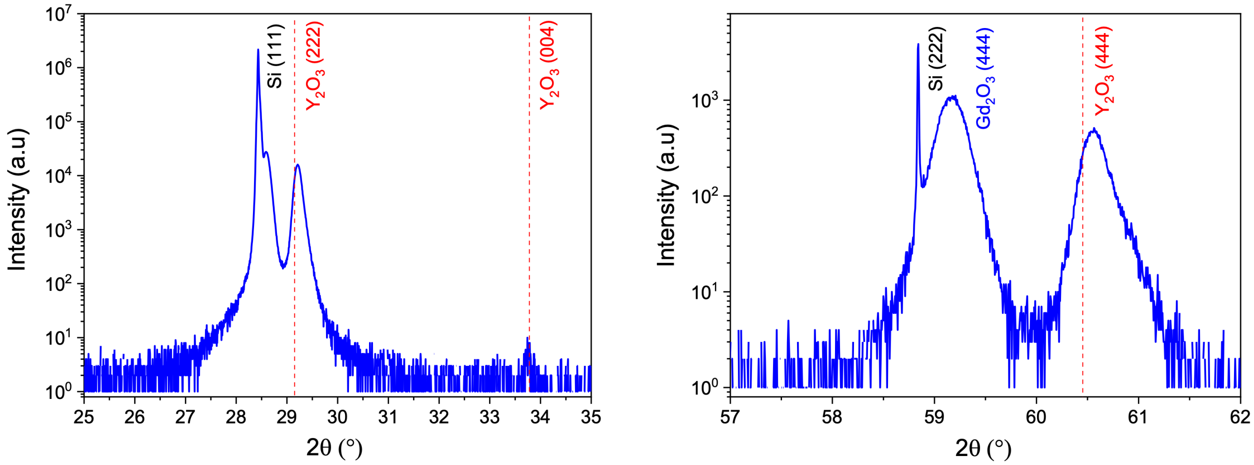

Fig. S 3: $\theta/2\theta$ scan at low (left) and high (right) angles confirming a single out-of-plane orientation for the Y_2O_3: Eu^{3+} thin film.

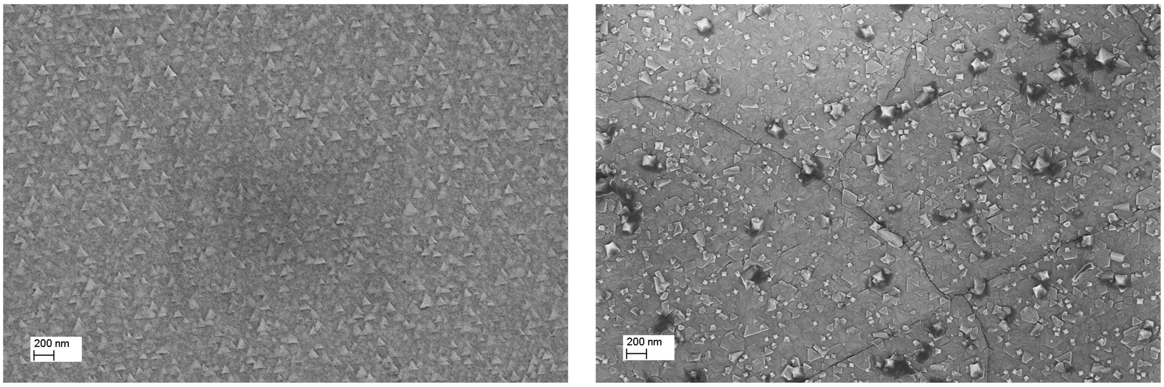

Fig. S 4: Scanning electron microscopy images of the (left) Gd_2O_3(111)/Si(111) substrate, acquired before CVD deposition, and (right) CVD Y_2O_3: Eu^{3+} thin film.

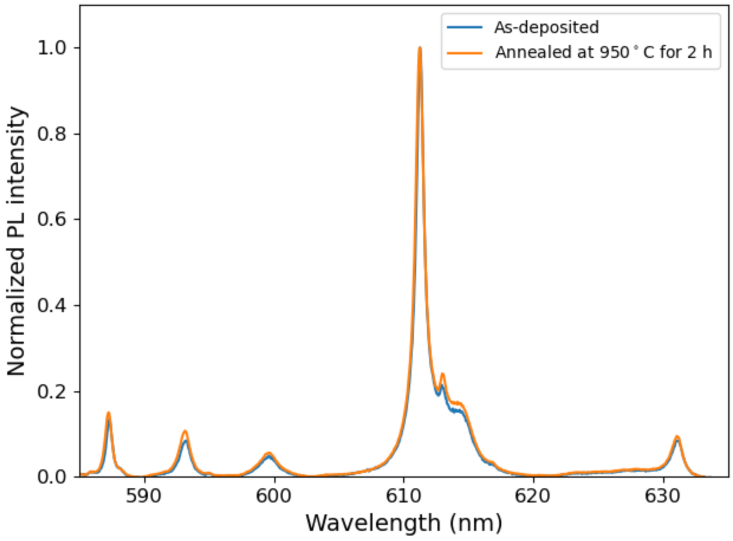

Fig. S 5: Eu^{3+} emission spectra recorded at room temperature from the as-deposited film and after annealing at 950 °C for 2 h. No difference is observed, confirming the absence of parasitic phases after annealing.

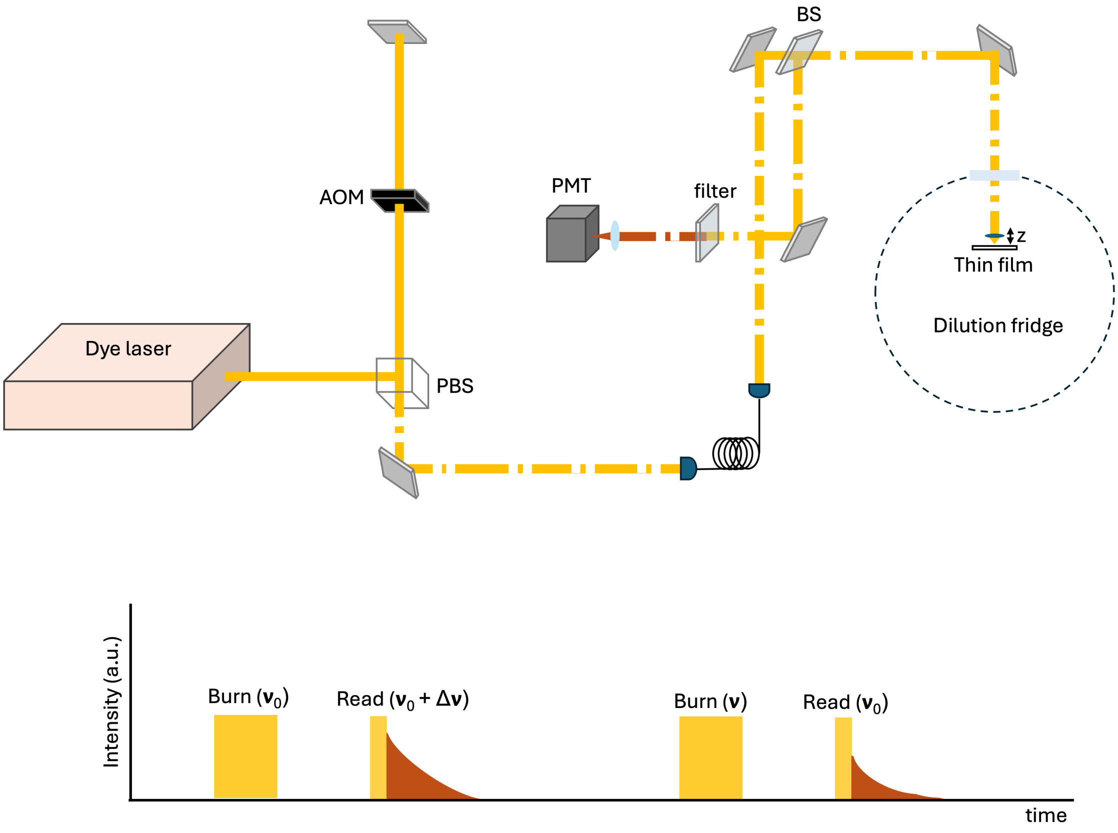

Fig. S 6: (Upper) Experimental setup. AOM stands for acousto-optic modulator, PBS for polarizing beam splitter, PMT for photomultiplier tube, and BS for beam splitter. The CW output of the laser is modulated by the AOM yielding the spectral hole burning sequence, represented by the discontinued line in the drawing. (Lower) Schematic view of the pectral hole burning sequence. The time-resolved detection focuses on the Eu^{3+} emission following the read pulse, stronger when probing the ions spectrally detuned from the spectral hole than when probing the ions left within the spectral hole.
